# Maturing iPSC-Derived Cardiomyocytes

**DOI:** 10.3390/cells9010213

**Published:** 2020-01-15

**Authors:** Bor Luen Tang

**Affiliations:** 1 Department of Biochemistry, Yong Loo Lin School of Medicine, National University of Singapore, Singapore 117596, Singapore; bchtbl@nus.edu.sg; Tel.: +65-6516-1040; 2 NUS Graduate School for Integrative Sciences and Engineering, National University of Singapore, Singapore 119077, Singapore

Cardiovascular disease is a major cause of mortality worldwide. The advent of human-induced pluripotent stem cells (hiPSCs) technology have enabled the reliable generation of individual-derived cardiomyocytes (CMs) [1]. Together with improvements in CM isolation techniques in culture [2] and the increased scalability of CM production from hiPSCs [3], hiPSC-CMs hold great promises in cardiopathological disease modelling and autologous tissue transplantation. However, as it is widely known, hiPSC-CMs more closely resemble CMs in the embryonic or fetal stages, and are immature in terms of marker expressions, electrophysiological properties, ultrastructural features and metabolic signature. Pertaining to this last feature, immature CMs prefer to use glycolysis for their energy metabolism, as opposed to β-oxidation of fatty acids [4]. This immature phenotype of newly derived hiPSC-CMs limits their immediate applications, and a prolonged culture period necessary for better maturation is neither logistically nor economically ideal. A number of strategies have been employed to hasten the maturity of hiPSC-CMs, which includes the addition of thyroid hormone T3, the bioengineering of culture environment or scaffolds, the application of mechanical stimuli [5,6] and other genetic/epigenetic manipulations [7].

In their recent report in *Cells*, Horikoshi and colleagues [8] differentiated hiPSCs into CMs using a rather standard protocol involving glycogen synthase kinase-3 and Wnt signaling inhibitors, and purified troponin T-positive CMs using a lactate-containing medium [2]. The authors then switched the hiPSC-CMs into either a control medium with glucose (RPMI 1640 with B-27 supplement) or a “maturation” media (DMEM with no glucose, supplemented with amino acids, insulin, transferrin, selenium solution, taurine and some components of B27) that contained linoleic acid/oleic acid with albumin. Compared to hiPSC-CMs kept for seven days in the control medium, those cultured in the maturation medium adopted a more prolonged, rod-like morphology, and their more organized troponin T staining pattern exhibited sarcomere-type striations. Ultrastructural analyses showed that these cell clusters also have better organized myofibrils, with a visibility of muscle fiber Z-lines that are seemingly aligned with those of adjacent fibers. Importantly, these have a larger number of mitochondria associated with the forming myofibrils. Gene expression profiling also indicated that hiPSC-CMs cultured in the maturation media have higher levels of mature CM-related genes, including those encoding ion channels and the cardiac ryanodine receptor RYR2 (which regulates sarcomeric Ca^2+^), as well as the key metabolic transcription regulator peroxisome proliferator-activator receptor alpha (PPARα) (a major regulator of genes involved in fatty acid β-oxidation). Thus, in terms of morphology and transcript profile, hiPSC-CMs cultured in the fatty acid-enriched maturation media appeared much more “sarcomerically” mature than those kept in a glucose-based media.

How did the above translate into the metabolic capacity of the mature hiPSC-CMs? The authors measured and showed that the mature hiPSC-CM populations have a significantly higher oxygen consumption rate (OCR), indicating that these cells have either an increased mitochondrial maturity or an enhanced mitochondrial oxidative capacity. Furthermore, when cells were given palmitate, mature hiPSC-CMs displayed a larger change than the control hiPSC-CMs in both basal and maximal OCR. This palmitate-induced OCR in matured hiPSC-CMs is abolished by the fatty acid oxidation inhibitor etomoxir, but not by 2-deoxyglucose inhibition of glycolysis. Matured hiPSC-CMs thus appear much more capable of utilizing exogenous palmitate for energy than the control iPSC-CMs. Interestingly, assessments of glycolytic flux parameters via measurements of the extracellular acidification rate (ECAR) indicated that ECAR levels in terms of glycolysis, glycolytic capacity, and glycolytic reserve were all significantly higher in mature hiPSC-CMs. These mature hiPSC-CMs, despite their fatty acid utilizing capability, are thus not impaired in terms of glycolytic capacity, and could readily switch to glucose as an energy substrate when necessary.

The findings of Horikoshi and colleagues, as the authors claimed, showed that fatty acid-containing maturation medium can promote hiPSC-CMs to undergo maturation [8]. As the supplements used in the control and maturation medium are not equal, the authors were careful in pointing out the caveat of potential maturation enhancing roles by taurine, carnitine, and selenium. However, the authors’ findings are indeed very much in line with a flurry of other recent reports indicating that fatty acids could enhance hiPSC-CMs maturation [9,10,11]. That a simple substitution of fatty acids for glucose in the culture medium could have such a significant maturation effect on hiPSC-CMs is certainly an encouraging advance. Clearly this change is easier and cheaper to implement compared to methods involving sophisticated scaffold engineering, constant mechanical stimulation, or complex genetic/epigenetic manipulations. A question that was not pointedly addressed by Horikoshi and colleagues in their report is the underlying mechanism as to how a fatty acid-enriched, no-glucose medium could induce hiPSC-CMs maturation. PPARα is known to play a critical role in cardiomyocyte maturation, and PPARα agonists have been shown to promote cardiomyocyte maturation [12,13]. Furthermore, glucose is known to suppress the expression of PPARα [14]. As a fatty acid sensing transcriptional regulator [15], PPARα’s induction and activity by added fatty acids could thus conceivably underlie the transformation of immature iPSC-CMs towards a more mature phenotype.
